# Supplementary figures and images for: Evolutionary origins of the emergent ST796 clone of vancomycin resistant Enterococcus faecium
Source: PeerJ. 2017 Jan 24;5:e2916. doi: 10.7717/peerj.2916 (PMC5267571; doi:10.7717/peerj.2916)

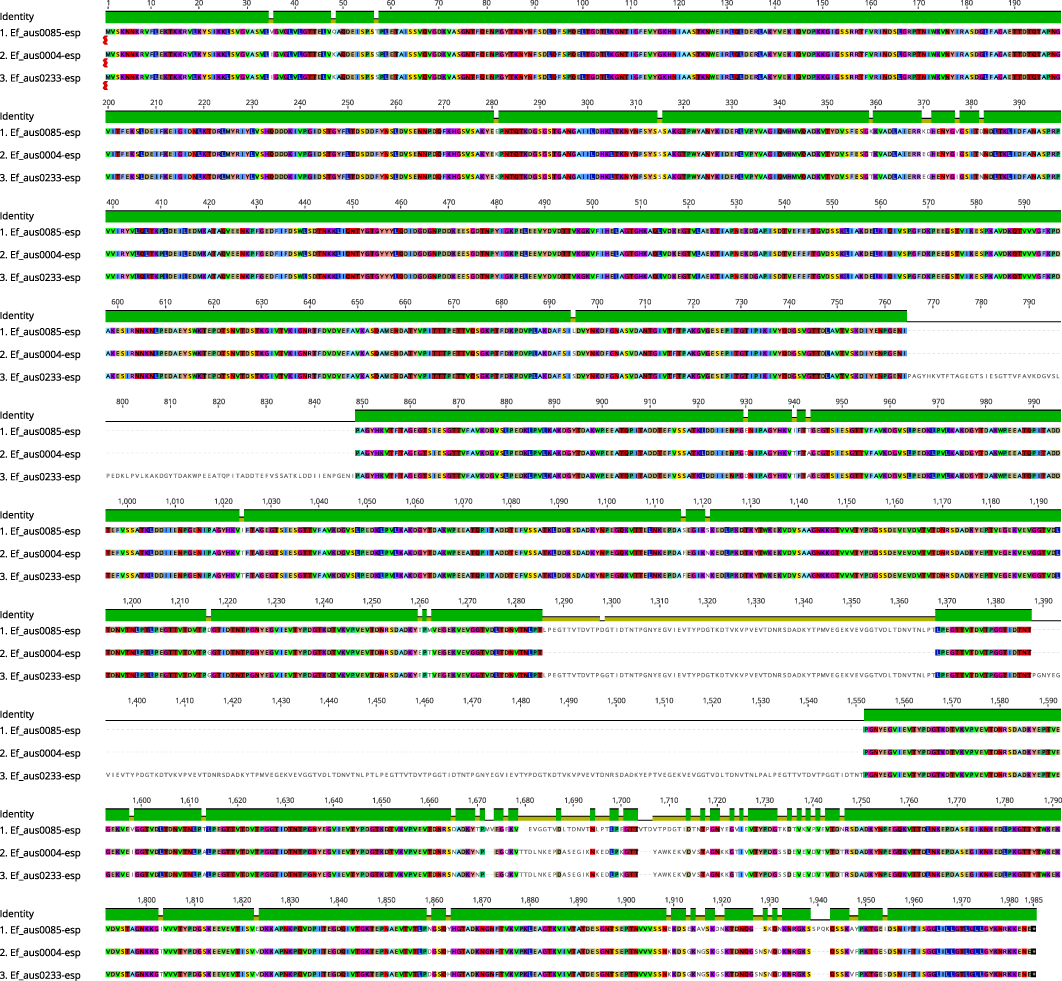

Supplement: Figure S1 — MUSCLE alignment of Esp orthologs among Ef_aus004, Ef_aus0085 and Ef_aus0233. [file peerj-05-2916-s001.png]

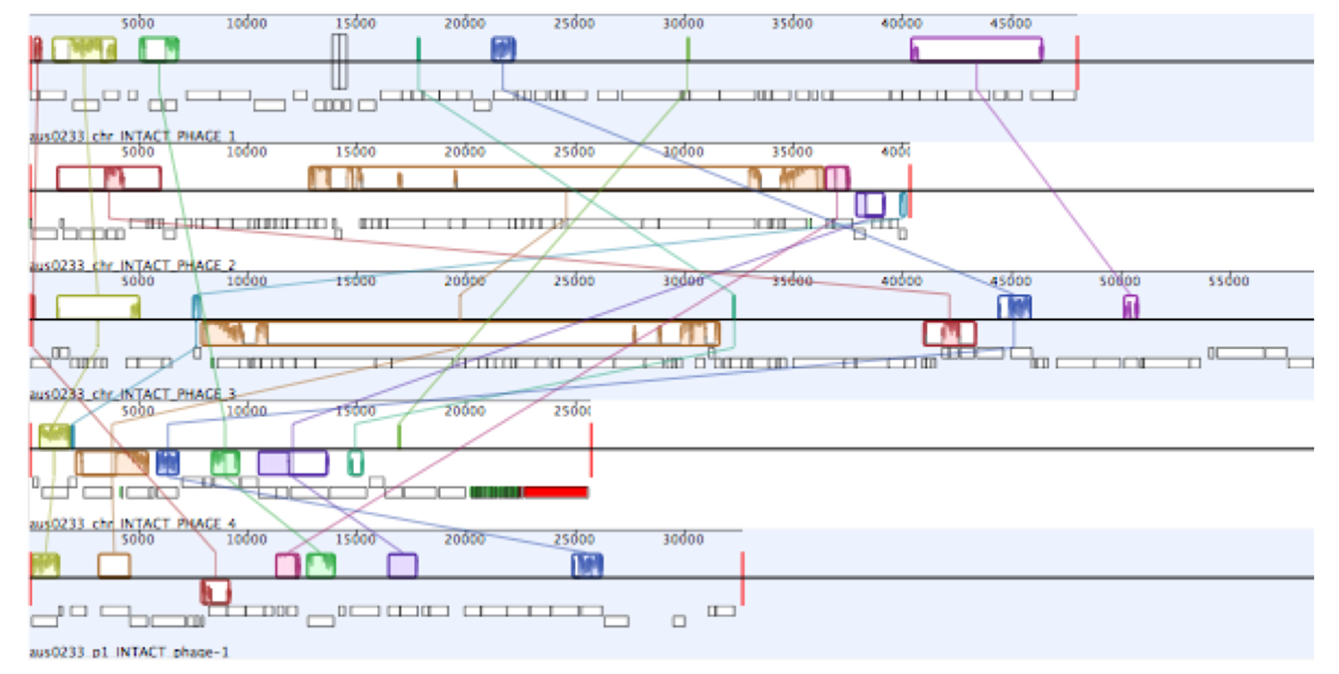

Supplement: Figure S2 — Mauve alignment of prophages detected in the Ef_aus0233 genome [file peerj-05-2916-s002.png]
